# Effectiveness of selective area growth using van der Waals h-BN layer for crack-free transfer of large-size III-N devices onto arbitrary substrates

**DOI:** 10.1038/s41598-020-77681-z

**Published:** 2020-12-10

**Authors:** Soufiane Karrakchou, Suresh Sundaram, Taha Ayari, Adama Mballo, Phuong Vuong, Ashutosh Srivastava, Rajat Gujrati, Ali Ahaitouf, Gilles Patriarche, Thierry Leichlé, Simon Gautier, Tarik Moudakir, Paul L. Voss, Jean Paul Salvestrini, Abdallah Ougazzaden

**Affiliations:** 1 Georgia Tech Lorraine, UMI 2958, Georgia Tech-CNRS, 2 rue Marconi, 57070 Metz, France; 2 Georgia Institute of Technology (School of Electrical and Computer Engineering), UMI 2958, Georgia Tech-CNRS, Atlanta, GA 30332-0250 USA; 3 CNRS, UMI 2958, Georgia Tech-CNRS, 2 rue Marconi, 57070 Metz, France; 4 Centre de Nanosciences et de Nanotechnologies, Université Paris-Saclay, C2N-Site de Marcoussis, Route de Nozay, 91460 Marcoussis, France; 5 Institut Lafayette, 2 rue Marconi, 57070 Metz, France

**Keywords:** Electronic devices, Two-dimensional materials, Two-dimensional materials

## Abstract

Selective Area van der Waals Epitaxy (SAVWE) of III-Nitride device has been proposed recently by our group as an enabling solution for h-BN-based device transfer. By using a patterned dielectric mask with openings slightly larger than device sizes, pick-and-place of discrete LEDs onto flexible substrates was achieved. A more detailed study is needed to understand the effect of this selective area growth on material quality, device performance and device transfer. Here we present a study performed on two types of LEDs (those grown on h-BN on patterned and unpatterned sapphire) from the epitaxial growth to device performance and thermal dissipation measurements before and after transfer. Millimeter-size LEDs were transferred to aluminum tape and to silicon substrates by van der Waals liquid capillary bonding. It is shown that patterned samples lead to a better material quality as well as improved electrical and optical device performances. In addition, patterned structures allowed for a much better transfer yield to silicon substrates than unpatterned structures. We demonstrate that SAVWE, combined with either transfer processes to soft or rigid substrates, offers an efficient, robust and low-cost heterogenous integration capability of large-size devices to silicon for photonic and electronic applications.

## Introduction

Hexagonal boron nitride (h-BN)-based mechanical transfer technique have emerged as a promising technology for heterogenous integration of III-Nitride devices and their application for the rapidly growing market of wearable, flexible electronics and internet of things (IoT)^1^. This technology has been accelerated through recent progress in the epitaxial growth of layered h-BN and the subsequent van der Waals (vdWs) epitaxy of III-Nitride materials by metal organic vapor phase epitaxy (MOVPE) technique^2–7^. Recent reports have been made of transfer of some key opto-electronic devices (HEMTs, LEDs and solar cells) to flexible and rigid substrates^8–10^. A simple lift-off of pre-diced devices has also been demonstrated^11^. This technique consists of patterning the epi-wafer surfaces using a dielectric mask so that growth selectively occurs in millimetric areas. This selective area van der Waals epitaxy (SAVWE) enabled a pre-die separation before the growth and allowed the transfer on tape of LEDs without dicing.

All of these experiments showed the large potential of the layered h-BN-based mechanical transfer technique on different host substrates in terms of (i) thermal dissipation that improve the efficiency of HEMT transistors when using a SiC substrate^12^, (ii) thermal confinement that allows for higher sensitivity and shorter time-response HEMT-based gas sensor when using copper tape as substrate^8^ (iii) device flexibility when using plastic substrate^9,10^, and (iv) better reusability of the growth substrate and a faster release rate when compared to conventional lift-off techniques, namely laser lift-off^13^ and chemical selective etching^14^.

On the other hand, these reported experiments revealed two critical weaknesses of wafer scale vdWs hBN for device transfer purpose. The first one is related to the delamination of the h-BN and 3D structures after epi growth^7,8^. This could be explained by the fact that for large area substrates the difference between the thermal expansion coefficients between the 3D heterostructures and the BN/sapphire substrate doesn’t result in creation a wafer bow as in the case of GaN grown on sapphire substrate^15^ but on spontaneous and sporadic delamination of the heterostructure from the substrate during the cooling process after the epitaxial growth or during the device fabrication steps^7,8^. The second issue is the cracks which appear in the devices and are induced during the lift-off and transfer process, leading to a low fabrication yield which further decreases with the size of the transferred devices^1,12^. This effect is mostly caused by the mechanical damages resulting from the lift-off and transfer process that required delicate manipulation. As a result, transferred layers came with a high crack density randomly distributed over the surface which limited the size of crack-free devices to only a few hundreds of squared microns.

In order to study the potential of SAVWE to address these issues, we have performed a side-by-side study of LEDs grown on layered h-BN with and without SAVWE. A benchmarking and testing of all the process steps from epitaxy, to device fabrication, lift-off, and transfer of devices have been performed. For these purposes, millimetric size LEDs were fabricated on patterned and unpatterned sapphire substrates and then released from their native substrates and transferred to different host substrates.

## Results

### Epitaxial growth and material characterization of MQWs InGaN-based LEDs heterostructures

**Figure 1:**
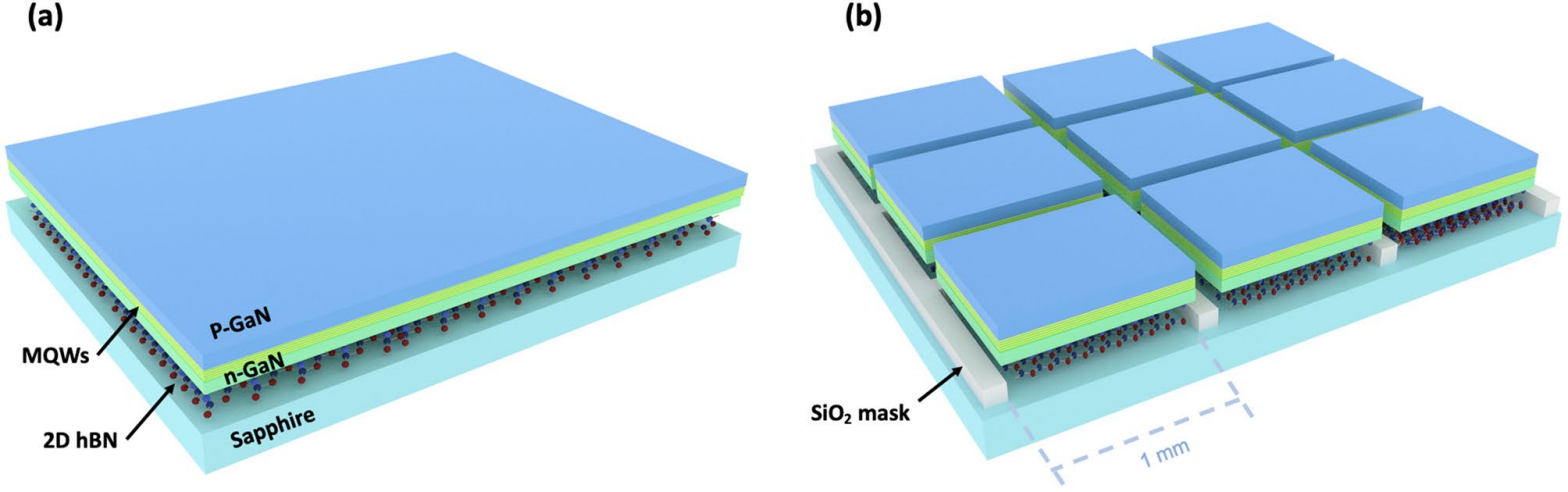
Schematics of the two grown structures: (**a**) LEDs on h-BN/unpatterned sapphire, (**b**) LEDs on h-BN/patterned sapphire with SiO_2_ mask.

Two sets of LEDs heterostructures have been grown by MOVPE. The core of the active region of the LED heterostructures is the same for both sets and consists of a 200-nm-thick Al_0.14_Ga_0.86_N layer used as buffer, 175-nm-thick magnesium-doped GaN layer, 5 In_0.15_Ga_0.85_N (2.5 nm)/GaN (12 nm) quantum wells, and a 300-nm-thick silicon-doped GaN layer. One set was grown on epi-ready 2″ sapphire substrates covered with 3 nm h-BN for vdWs epitaxy and as a mechanical release layer for LEDs transfer. The second set of LEDs was grown by using the SAVWE approach. It consists of using 2″ sapphire substrates patterned with SiO_2_ masks and covered with 3 nm BN layer. In this approach, the localized vdWs epitaxy reduces the 3D heterostructures delamination, since they are localized to relatively small unmasked area of 1 mm^2^ to 1 cm^2^ and separated from each other. The details of the approach have been reported elsewhere^11^. In addition, the SAVWE makes the transfer easier since after process fabrication, devices are pre-diced and can be individually lifted-off and placed on an arbitrary substrate. The buffer on both structures consists of a 200 nm thick AlGaN layer. The schematic of the 2 epitaxial structures grown on unpatterned and patterned sapphire substrates are shown on Fig. 1. Both sets of structures were grown in the same runs (see section “Methods” for details).

**Figure 2:**
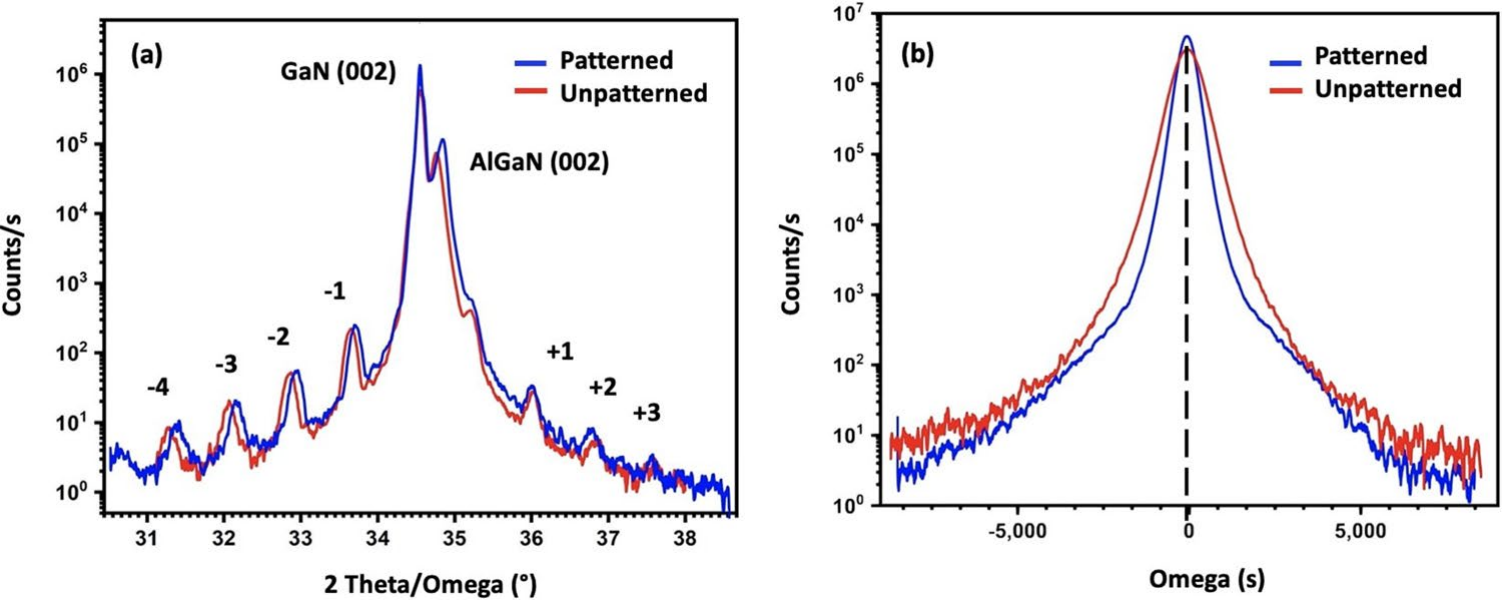
(**a**) High resolution X-ray diffraction 2θ − ω scans of the two grown samples. (**b**) High resolution X-ray diffraction ω-scans of the two grown samples along the GaN (0002) reflections.

In depth structural characterizations have been conducted after the growth. Figure 2a shows HR-XRD 2θ − ω scans of the two heterostructures. Both X-ray diffraction spectra are comparable. One can observe an intense GaN peak and several satellite peaks from the MQWs up to the fifth order with Pendellosung fringes indicating good periodicity and abrupt interfaces in the quantum wells. GaN (0002) and AlGaN (0002) diffraction peaks are also observed. Interestingly, the GaN (0002) ω-scans, depicted in Fig. 2b, shows smaller full-width at half-maximum (FWHM) for the patterned structure (1044 arcsec) than the one of the unpatterned heterostructure (1584 arcsec) indicating better crystalline quality since in wurtzite GaN films the broadness of symmetric (0002) ω-scan spectra is mainly related to the presence of dislocations.

**Figure 3:**
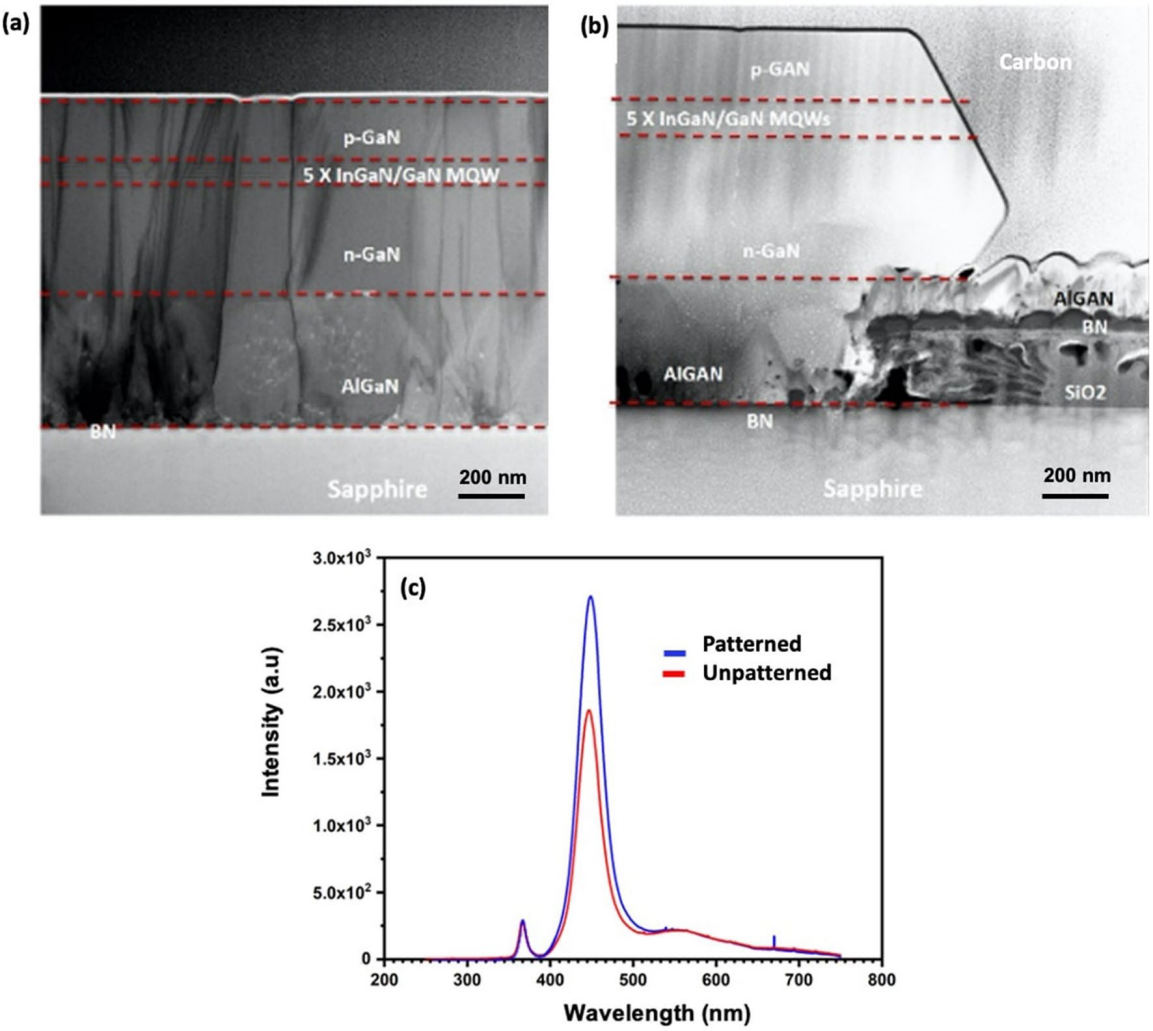
(**a**) TEM image of the unpatterned sample, (**b**) TEM image of the patterned sample, (**c**) cathodoluminescence spectra at 8 kV recorded on both structures at room temperature. Both spectra have been normalized with respect to their GaN peak intensity.

This is confirmed by the TEM analysis of both types of structures. As shown in Fig. 3a, a significant reduction of the threading dislocation density is observed in the patterned structure compared to the unpatterned structure (Fig. 3b). These can be explained by the mechanism of the growth of 3D layers on 2D crystals. Indeed, since both structures were grown on hBN surface having no dangling bonds, the threading dislocations are mostly originating from the AlGaN islands formation during the nucleation layer on h-BN. SAVWE likely promotes the growth of large AlGaN and h-BN islands leading to the lateral coalescence with less generation of stacking faults and dislocations than in the case of the growth on unpatterned substrate.

Optical characteristics of the MQW structures have been investigated, as shown in Fig. 3c. For that, cathodoluminescence (CL) spectra of both structures were recorded at room temperature with an electron beam excitation energy of 8 keV and normalized with respect to the GaN CL peak intensity. For both structures a MQW emission peak around 445 nm has been obtained which corresponds to 14% In content in the quantum wells. The corresponding peak intensity is significantly higher for the patterned structure (× 1.5) than the one of the unpatterned structures. This result is in good agreement with TEM and X-ray diffraction data and can be expected since the threading dislocations act as nonradiative recombination centers and impact the luminescence.

### Fabrication and transfer of LEDs to arbitrary substrates

**Figure 4:**
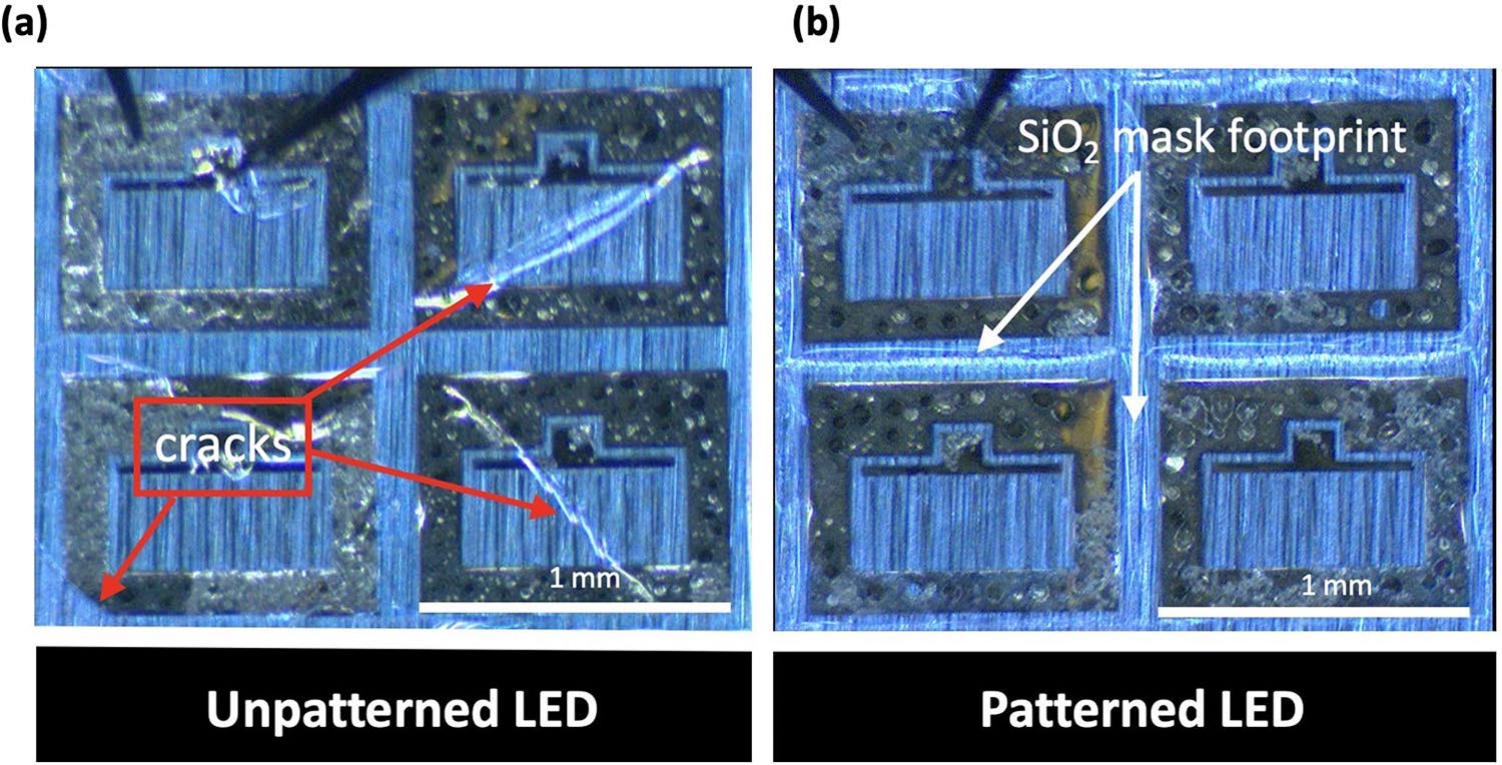
Optical microscope images of large area LEDs released and transferred to aluminum tape from (**a**) unpatterned and (**b**) patterned structures. Several cracks are observed for devices released from the unpatterned structure.

The fabrication of the LEDs was based on a standard photolithography process (see section “Methods” for details). The fabricated LEDs had an area of 1 mm × 0.5 mm with n-contact surrounding the device and 16 fingers for the p-contact. After fabrication of LEDs on both structures, few mm^2^ surface areas containing four LEDs were released by mean of water-soluble tape through a vertical lift-off that minimizes induced shear strains in the device layers and transferred from the two structures to an aluminum tape containing a 5-μm-thick acrylic adhesive layer. Figure 4a,b show microscope images of transferred unpatterned and patterned LEDs, respectively. The transferred LEDs from the patterned structure were crack-free while those transferred from the unpatterned structure showed large cracks crossing half of the devices. Analysis was conducted on 20 released LED devices from each sample. 18 functional devices without cracks were obtained on patterned devices while 6 crack-free LEDs were obtained on unpatterned devices. This results correspond to a yield increase from 30 to 90% and are another proof of efficiency of SAVWE approach.

**Figure 5:**
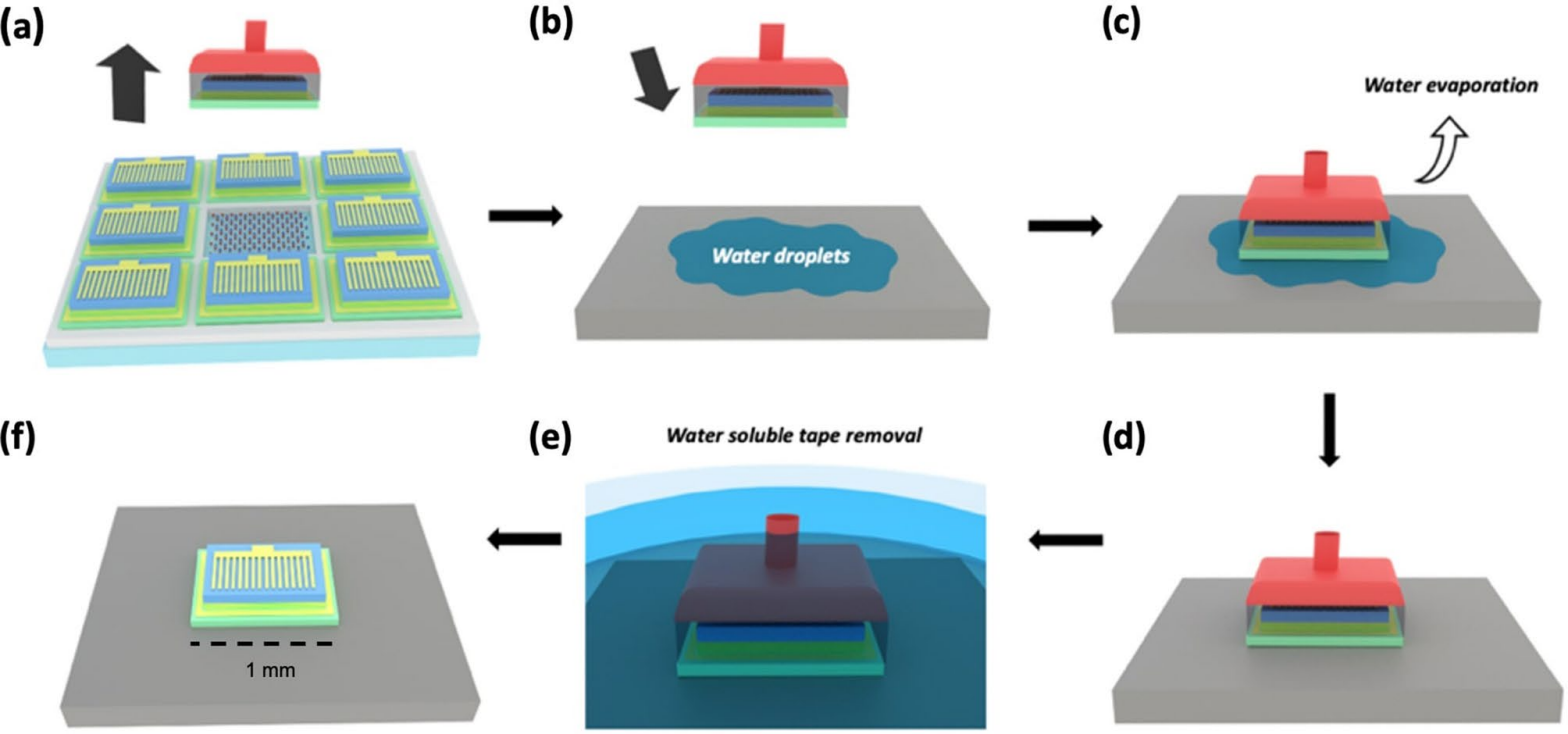
Schematics of the lift-off and van der Waals bonding transfer on rigid substrate. (**a**) Vertical release from hBN/sapphire native substrate (**b**) water droplets deposition of silicon (**c**) deposition of released LEDs onto silicon (**d**) vdWs bonding on silicon after water evaporation (**e**) water soluble tape removal (**f**) LED transferred to silicon.

Subsequently, device transfer to silicon rigid substrate was attempted. For efficient comparison, slightly smaller (1 mm × 0.5 mm) LEDs were considered to avoid too many cracks in devices transferred from the unpatterned structure. This transfer on rigid and non-sticking substrates was achieved using water capillary force and without the use of an intermediary adhesive layer. First, a small droplet of water was deposited on the host substrate, then the mechanically released LEDs were applied on the receiving substrate. As the water evaporates, it pulled the surfaces together by capillary action until a robust and uniform vdWs bonding was obtained. The top tape was then dissolved in water and the transferred devices were cleaned by a flow of acetone and isopropanol to remove any remaining residues. The process flow illustration of this transfer is given in Fig. 5.

**Figure 6:**
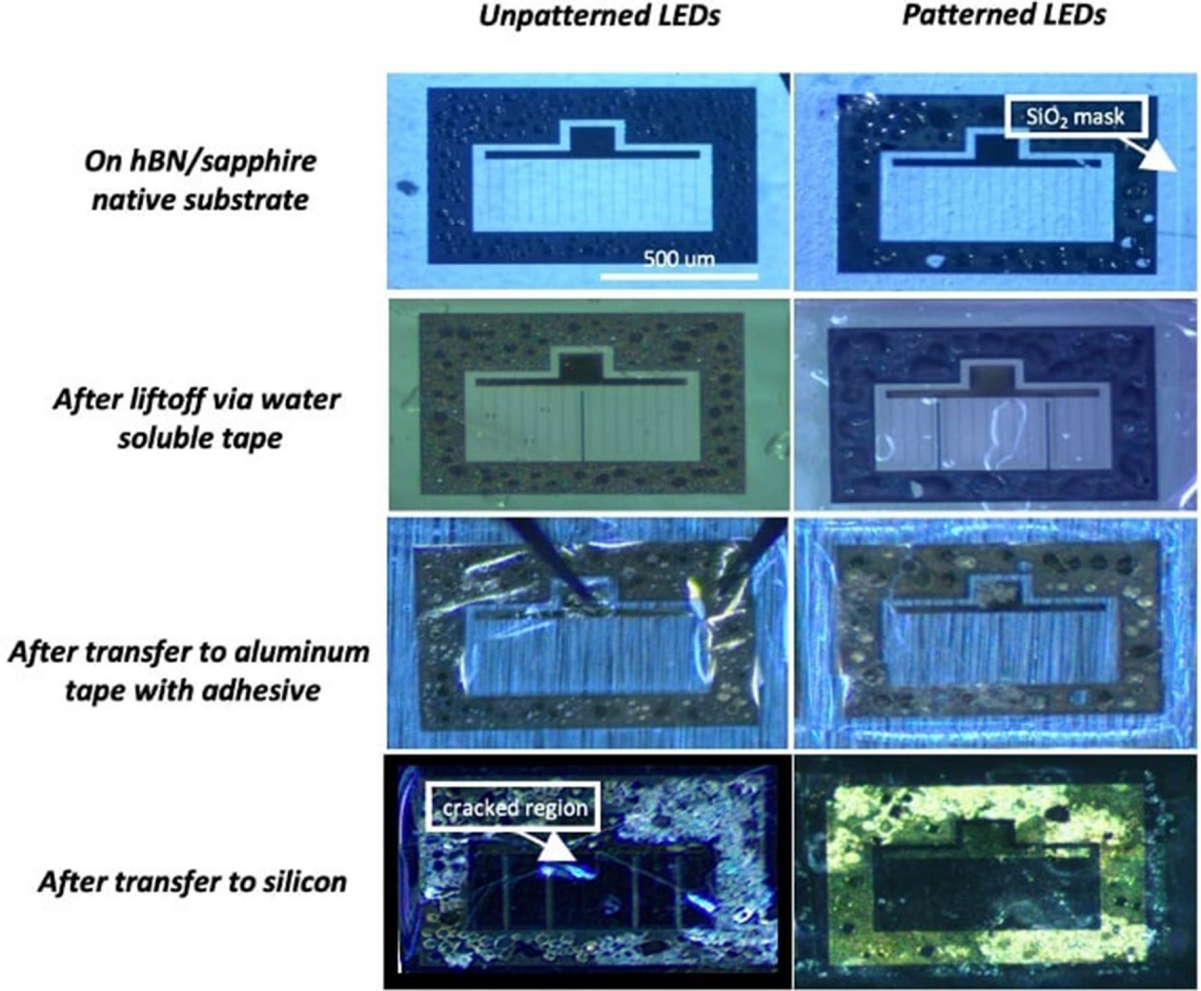
Microscope images of the devices taken at the different steps of the transfer process for both unpatterned and patterned structures: after the fabrication process, lift-off, and transfer on aluminum tape and silicon substrate.

Figure 6 shows representative microscope images of the devices taken at the different steps of the transfer process for both unpatterned and patterned structures: after the fabrication process, lift-off, and transfer on aluminum tape and silicon substrate. As it can be seen, the lift-off step is achieved without any cracks whatever the structures. It is also the case for the transfer to aluminum tape, even if the yield is a bit higher for the devices transferred from the patterned structure. Surprisingly, we were not able to transfer crack-free devices on silicon substrate from the unpatterned structure while we achieved systematically crack-free transfer from the patterned one. This will be investigated in detail in a further study.

### Electro-optical characterization of LEDs before and after transfer

**Figure 7:**
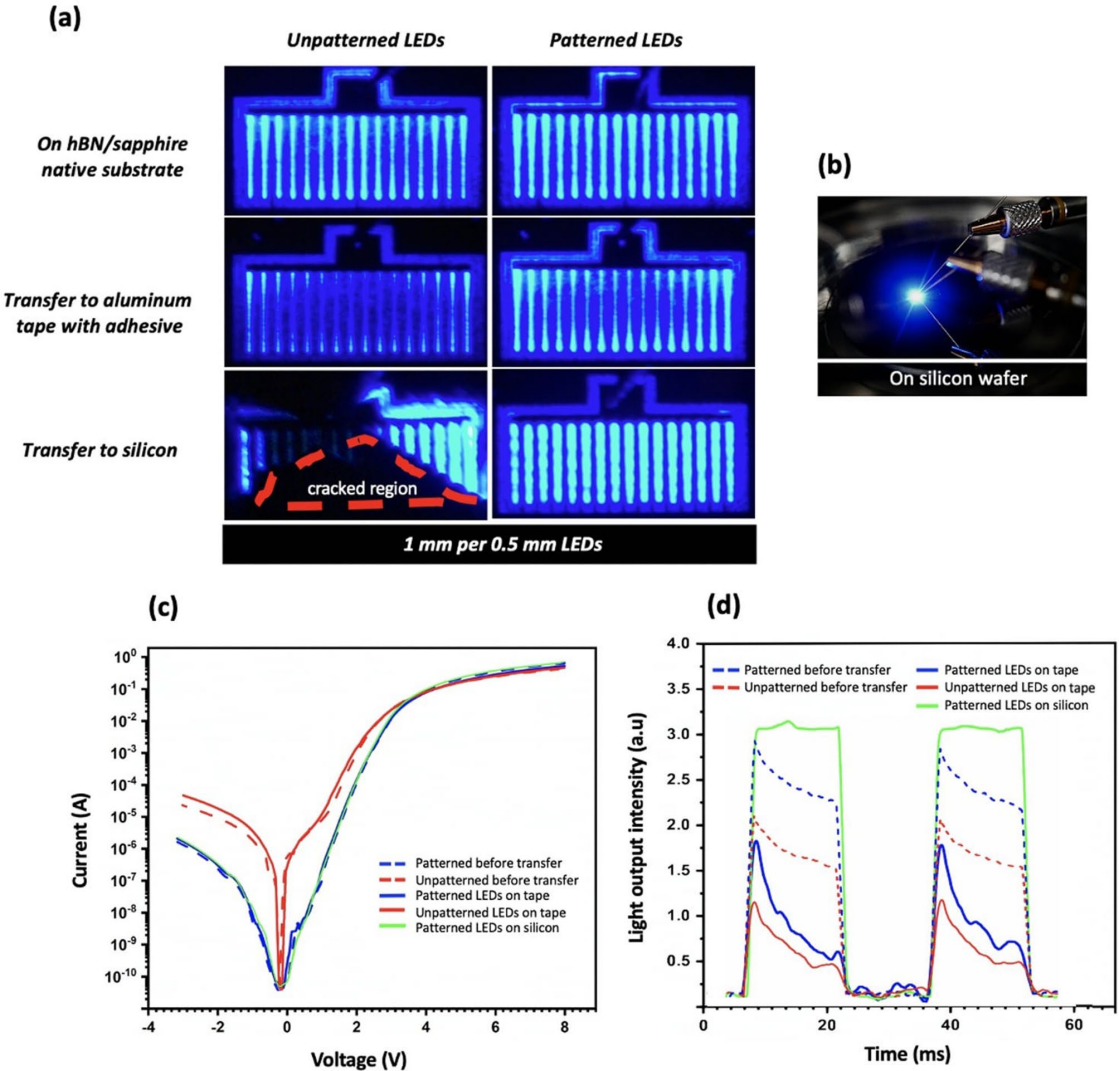
(**a**) EL images of LEDs before and after transfer to different substrates (**b**) Photograph of light emission from an LED on silicon (**c**) I–V characteristics of LEDs before and after transfer to aluminum tape and silicon (**d**) Light output versus operation time of patterned and unpatterned devices before and after transfer to aluminum tape and silicon.

Figure 7a shows electro-luminescence images of the LEDs from both structures recorded, for a pulsed (15 ms duration, duty cycle of 0.5) electric current of 100 mA, before and after the fabrication process and transfer on aluminum tape (for reference) and silicon substrate. Figure 7b is a photograph of light emission from an LED on silicon. In Fig. 7a, one can see that for devices transferred to aluminum tape, the luminescence is relatively higher in the case of the patterned LEDs. Moreover, in both structures the center of the LEDs looks darker than the edges. We attributed this attenuation to the current induced heating and poor thermal dissipation due to the acrylic layer of the tape which has a low thermal conductivity. It is to be noticed that this effect is more pronounced in the case of the unpatterned LEDs. This could be related to the high density of dislocations in the unpatterned structures as it is well known that the threading dislocation lines have an adversely effect on the thermal conductivity of III-N semiconductors because of the phonon-dislocation scattering mechanism^16^. Also it has been reported that micro-cracks in the materials reduces significantly the thermal conductivity because the heat transfer by radiation or convection across the cracks is completely absent^17^.

Homogenous and more intense electro-luminescence was obtained for patterned LEDs transferred to silicon substrate. This is a clear evidence of the good vdWs bonding between the device and the silicon substrate which allows for an efficient thermal dissipation thanks to the relatively high thermal conduction of the silicon. Electro-luminescence was obtained also in the unpatterned device transferred on silicon substrate. Nevertheless, due to the cracks in the devices, current was able to flow only in a small part of the device, leading to high local current density and electro-luminescence. In addition, quantitative electro-optical characterization was conducted through pulsed current–voltage measurements on LEDs before and after transfer. Results are shown in Fig. 7c. The data of the unpatterned LED on silicon were not reported because of its leaky behavior likely due to the cracked area. All the other devices exhibited a turn-on voltage around 3 V. Clearly, unpatterned LEDs showed degraded behavior with reverse leakage current more than one order of magnitude larger than in the case of patterned LEDs. Forward current below the turn-on voltage due to recombination in the depletion region is also two order of magnitude higher. It is also to be noticed that patterned LEDs showed very little variation of their I–V before transfer and after transfer on either aluminum tape or silicon substrate. On the contrary, unpatterned LEDs came with larger alteration, such as higher reverse leakage and lower forward current of the I–V behavior.

The role of the thermal dissipation in the inhomogeneity of the electro-luminescence of the different LEDs is clearly evidenced in Fig. 7d showing the time dependence of the emitted optical power of the patterned and unpatterned LEDs before and after transfer and recorded for a pulsed (15 ms duration, duty cycle of 0.5) electric current of 100 mA. These data confirmed what was qualitatively observed on Fig. 7a. First, the LEDs transferred on aluminum tape show some degradation of the output optical power which is even more pronounced in the case of the unpatterned LEDs. Second, except for the patterned LEDs transferred on silicon for which the optical power showed a plateau-like behavior during the full duration of the current impulse, the other LEDs before and after transfer exhibited a decay of the optical power after a few hundred of μs and a significantly reduced peak intensity for the LEDs transferred on tape when compared to LEDs on hBN/sapphire and transferred on silicon. This indicates that thermal effects occur during the first microseconds of operation. This is clearly due to a lack of thermal dissipation induced by the host substrate since the decay is faster for LEDs transferred on aluminum tape than for LEDs before transfer and for LEDs transferred to silicon for which no decay is observed. The decay observed for the LEDs before transfer is a bit surprising because one can expect a large thermal dissipation of the sapphire substrate. The thermal dissipation efficiency in that case is likely degraded by the presence of the thin AlGaN buffer and h-BN layers which could introduce large thermal resistance at their interfaces.

## Discussion

The results obtained in this study and summarized in Table 1 show that the SAVWE of III-N device coupled with the h-BN based mechanical lift-off and transfer on a host substrate lead to a large improvement of the crystalline quality of the active layers by a significant reduction of the threading dislocation density and thus an enhancement of the electrical and optical device performance.

**Table 1:** Summary of the benefits brought by the use of patterned growth (SAVWE) for the transfer process of devices on host substrates.

	Crystalline quality of epilayers	Crack free devices after release	Transfer yield of fully functional devices	Device performance (I–V and EL)
Patterned (SAVWE)	Good	Excellent (no cracks)	More than 90%	High
Unpatterned	Fair	Medium (presence of cracks for large devices)	Less that 30%	Medium

**Figure 8:**
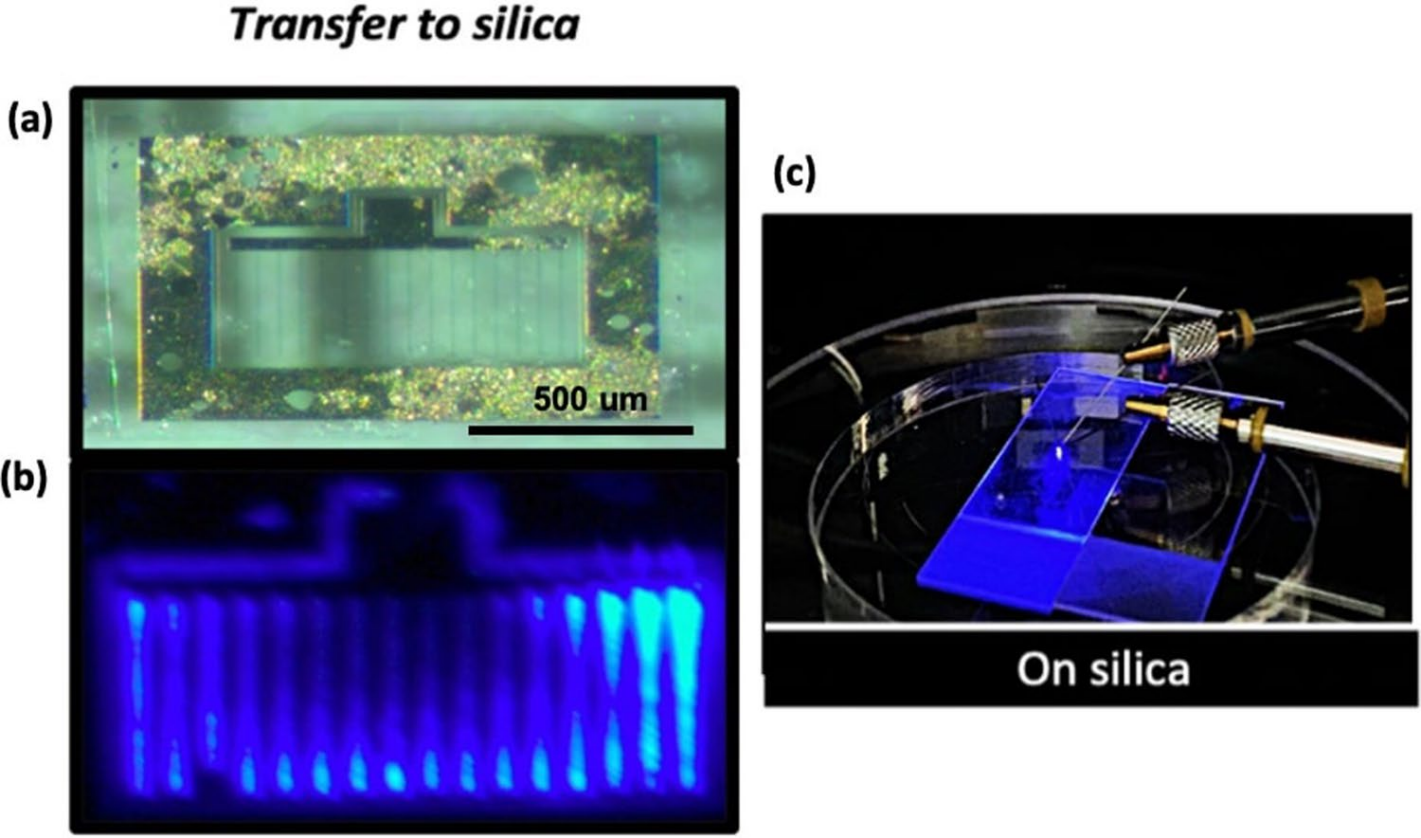
(**a**) Optical microscope image of a patterned LED transferred on a silica plate. (**b**) Corresponding electro-luminescence when a pulsed current is applied to the device. (**c**) Photograph showing the backside emission, guiding and decoupling of the light in the rough surface region of the plate.

Moreover, the yield of the transfer of large-size crack-free devices on host substrates is higher when using SAVWE. The technique developed in the frame of this study is also very useful for the efficient heterogeneous integration of III-N devices on CMOS compatible substrates. It can be easily extended to the transfer of other devices especially electronic devices and solar cells where the dimension of the devices could be quite large. Another application of such a process is the realization of waveguiding in III-N films which requires cladding layers of lower refractive index to confine light to the III-N layer. This could be achieved, much more easily than, for instance, the technique developed by C. Xiong et al.^18^*,* by transferring III-N layers to a SOI substrate where the silicon dioxide on top of the silicon wafer serves as one of this cladding layer. As a proof of concept and as shown in Fig. 8, we successfully transferred a crack-free patterned LED on a thick glass plate. One can notice that the back-side light-emission is guided in the glass plate owing to the perfect transparency of silica glass at the considered wavelength. Last, SAVWE could be very useful for the fabrication of devices with engineered shape such as triangle LEDs that allows for a better light-extraction^19^, this without any complicated dicing process.

## Methods

### Material growth and characterization

The epilayers were grown in an Aixtron MOVPE CCS 3 × 2″ reactor on (0001) 2-inch sapphire wafers. A 3 nm thick h-BN layer was first grown at 1300 °C. Then, an intermediate 200 nm thick AlGaN layer with an Al mole fraction of 14% was grown at 1100 °C. This layer acts as an interfacial buffer between sp3-bonded epitaxial films and layered h-BN and promotes nucleation. The LED structure comprises a 300 nm thick Si-doped GaN layer, 5-periods of InGaN/GaN multi-quantum wells (MQWs), and a 175 nm thick Mg-doped GaN layer. The 5 QWs structure consists of a 12-nm-thick GaN barrier layer and a 2.5-nm-thick InGaN QW layer with an In mole fraction of 15%. The electron and hole carrier concentrations in the Si- and Mg-doped GaN layers are 5 × 10^18^ and 1 × 10^17^ cm^−3^, respectively.

For the patterned substrate, a SiO_2_ mask was achieved by a photolithography-based process on an epi-ready sapphire substrate. 400 nm-thick SiO_2_ layer was deposited by plasma-enhanced chemical vapor deposition (PECVD) on a 2-inch sapphire wafer and the patterns were defined by optical lithography. The devices’ locations were opened by etching SiO_2_ by buffered oxide etch solution. Then, hBN and subsequent layers were grown similarly to the unpatterned substrate.

High resolution X-ray diffraction (HRXRD) scans were performed in Panalytical X’pert Pro MRD system with Cu K $\alpha$ radiation. The samples were prepared for STEM using focused ion beam (FIB) thinning and ion milling. 100 nm-thick carbon was deposited before FIB in order to protect the surface. Then, the high-angle Annular Dark Field Scanning Transmission Electron Microscopy (HAADF-STEM) was performed on an aberration-corrected JEOL 2200FS electron transmission microscope. Depth resolved cathodoluminescence (CL) spectra were measured with Horiba HR-320 system.

### Device processing and characterization

The fabrication of the LEDs follows the process described in Ref.^11^ using optical lithography. Dry etching was accomplished by Cl_2_/Ar inductively coupled plasma (ICP). Ti/Al/Ni/Au and Ni/Au layers, deposited by e-beam evaporation, were used for the n-contact and p-contact, respectively. The n-contact was annealed at 850 °C for 30 s under N_2_ and the p-contact was annealed at 600 °C for 60 s under O_2_. I–V measurements were achieved by an automated probe station. Electroluminescence images were taken by a CCD camera and optical emission versus time measurements were performed using a silicon photodiode connected to an oscilloscope.
